# Osteoblast Lineage Cells Can Discriminate Microscale Topographic Features on Titanium–Aluminum–Vanadium Surfaces

**DOI:** 10.1007/s10439-014-1108-3

**Published:** 2014-09-17

**Authors:** Rene Olivares-Navarrete, Sharon L. Hyzy, Mark E. Berg, Jennifer M. Schneider, Kelly Hotchkiss, Zvi Schwartz, Barbara D. Boyan

**Affiliations:** 1 Department of Biomedical Engineering, Virginia Commonwealth University, Richmond, VA USA; 2 Titan Spine LLC, Mequon, WI USA; 3 Wallace H. Coulter Department of Biomedical Engineering, Georgia Institute of Technology, Atlanta, GA USA; 4 Department of Periodontics, University of Texas Health Science Center at San Antonio, San Antonio, TX USA; 5 School of Engineering Virginia Commonwealth University, 601 West Main Street, Suite 331a, Richmond, VA 23284 USA

**Keywords:** Human mesenchymal stem cells, Osteoblast differentiation, Titanium alloy

## Abstract

Titanium (Ti) and Ti alloys are used in orthopaedic/spine applications where biological implant fixation, or osseointegration, is required for long-term stability. These implants employ macro-scale features to provide mechanical stability until arthrodesis, features that are too large to influence healing at the cellular level. Micron-scale rough Ti alloy (Ti–6Al–4V) increases osteoblastic differentiation and osteogenic factor production *in vitro* and increases *in vivo* bone formation; however, effects of overall topography, including sub-micron scale and nanoscale features, on osteoblast lineage cells are less well appreciated. To address this, Ti6Al4V surfaces with macro/micro/nano-textures were generated using sand blasting and acid etching that had comparable average roughness values but differed in other roughness parameters (total roughness, profile roughness, maximum peak height, maximum valley depth, root-mean-squared roughness, kurtosis, skewness) (#5, #9, and #12). Human mesenchymal stem cells (HMSCs) and normal human osteoblasts (NHOst) were cultured for 7 days on the substrates and then analyzed for alkaline phosphatase activity and osteocalcin content, production of osteogenic local factors, and integrin subunit expression. All three surfaces supported osteoblastic differentiation of HMSCs and further maturation of NHOst cells, but the greatest response was seen on the #9 substrate, which had the lowest skewness and kurtosis. The #9 surface also induced highest expression of α2 and β1 integrin mRNA. HMSCs produced highest levels of ITGAV on #9, suggesting this integrin may play a role for early lineage cells. These results indicate that osteoblast lineage cells are sensitive to specific micro/nanostructures, even when overall macro roughness is comparable and suggest that skewness and kurtosis are important variables.

## Introduction

Biomaterials such as titanium (Ti) and titanium alloys (titanium–aluminum–vanadium alloy [Ti6Al4V]) are used for many orthopaedic procedures due to their biocompatibility and mechanical properties.^2^,^13^,^24^ Macroscale roughened surfaces are incorporated into implant designs to stabilize the bone-implant interface and minimize micromotion during spinal fusion, also known as arthrodesis. These features are designed to create a mechanical interlock with the bone, but are too large in scale to affect the host response at a cellular level. In addition, these materials can be fabricated to have surfaces that are microtextured and a number of studies have shown that osteoblast lineage cells exhibit increased osteoblastic differentiation when they are cultured on Ti and Ti6Al4V substrates with microscale roughness in comparison to smooth surfaces.^15^,^16^,^21^,^26^ These *in vitro* results have been correlated with improved bone-to-implant contact and greater pullout strength *in vivo*.^5^,^22^,^23^

As industry strives to design implant surfaces that optimize cellular response, there is little information concerning the ability of these cells to discriminate between topographies of the same average micro-roughness, or whether cells at different osteoblast maturation states sense submicron- and nanoscale topographies differently. We previously compared the responses of MG63 osteoblast-like cells to electromachined Ti substrates with well-defined macro and microscale surface features (30 *µ*m diameter craters either adjacent to each other or spaced 30 *µ*m apart) overlain with submicron-scale structures with different nanoscale apices achieved by acid etching or oxidation.^27^ The results showed that the cells preferred the adjacent crater macrotopography and the micro/nanotopography achieved by acid etching, suggesting that submicron-scale and nanoscale surface features could influence outcomes, even when macro/microscale roughness was constant. Other studies using nanostructured surfaces confirm that cells can sense submicron features.^7^–^9^ However, these papers report results using well defined structured surfaces on test substrates including laser patterned tissue culture polystyrene (TCPS). To date, there has not been a careful analysis of macro-scale surfaces of comparable micro-scale roughness but very different submicro-scale/nanoscale topographies fabricated from clinically relevant materials like Ti6Al4V.

Human osteoblast-like MG63 cells exhibit a more differentiated phenotype when cultured on Ti and Ti6Al4V surfaces with an average 3D roughness (Sa) of 1.8–2.6 *µ*m than when grown on Ti and Ti6Al4V surfaces with Sa ranging from 0.09 to 0.3 *µ*m.^15^,^17^ This phenotype is characterized by high levels of osteocalcin production. In addition, cells produce high levels of osteoprotegerin, which inhibits osteoclast activity and transforming growth factor beta 1 (TGFβ1), which both promotes extracellular matrix formation and inhibits osteoclasts.^1^ Importantly, MG63 cells produce bone morphogenetic proteins together with their inhibitors,^15^ angiogenic factors,^18^ and anti-inflammatory cytokines,^11^ resulting in a microenvironment that favors bone formation over bone resorption.

Human mesenchymal stem cells (HMSCs) exhibit a differentiated osteoblast phenotype when grown on the same surfaces that support a mature osteoblast phenotype. They do so even in the absence of medium supplements such as beta-glycerophosphate and dexamethasone, which are commonly used in cultures grown on tissue culture polystyrene (TCPS).^16^,^17^ Signaling *via* alpha2beta1 (α2β1) integrins is required for both osteoblast-like cells and HMSCs to exhibit osteoblast differentiation and maturation on microstructured Ti and Ti–6Al–4V.^17^,^19^ Whether this is a general requirement on Ti surfaces of comparable average microroughness or is specific to a limited set of topographies is not known. Co-culture studies with human osteoblasts grown on microstructured Ti indicate that factors generated by the human osteoblasts can induce osteoblastogenesis in MSCs that are grown on TCPS.^16^ Thus, the effects of the surface on osteoblast differentiation can extend to cells that are not themselves on the biomaterial, making it important to determine if differences in the microtopography of an implant surface can impact cell response.

A typical measurement used to determine the roughness characteristics of a material is the average roughness of a two dimensional (2D) profile section, Ra.^10^ While this measurement can provide an adequate overall analysis of the material surface, it does not describe the variation in the peaks and valleys of the roughness and the proportion of the measurements. There may also be differences in the steepness of the peaks and width of valleys. Both parameters will alter the ability of cells to attach and spread on a surface. How these differences might influence their differentiation or production of growth factors and cytokines that mediate osteogenesis is not known.

## Methods and Materials

### Ti6Al4V Substrates

**Figure 1:**
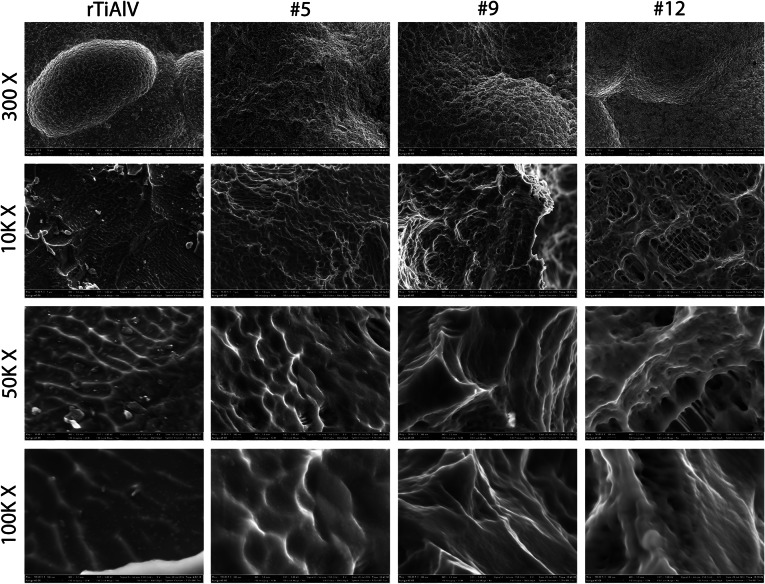
Scanning electron microscopy images of rough titanium alloy (rTiAlV) or substrates with modified topography (#5, #9, and #12). Images were taken at 300× (10 *μ*m scale bar), 10 k× (1 *μ*m scale bar), 50 k× (100 nm scale bar), and 100 k× (100 nm scale bar)

Ti6Al4V substrates (15 mm in diameter) were provided as a gift from Titan Spine LLC. Grit blasting, acid etching, or a combination of grit blasting and acid etching was used to generate a panel of surfaces. Some of the Ti6Al4V surfaces were exposed to additional secondary processing with variations of proprietary acid etch treatments, which retained the macro- and micro-scale features while creating finer submicron- and nano-scale features resulting in a macro/micro/nano (MMN) textured surface. Three of these surfaces, #5, #9 and #12 (Fig. 1) were found to have comparable Ra’s to previously reported double acid etched surfaces (rTiAlV, included here as a control)^15^  but different topographies as described below and were used for the cell culture experiments.

All disks were cleaned in an ultrasonic bath with detergent (Micro90 Soap, International Products Corporation, Burlington, NJ), rinsed in ultrapure water (Advantage A10; Millipore, Billerica, MA), and sterilized by autoclave (Model 2540E, Tuttnauer, Hauppauge, NY) before use.^20^

### Analysis of Surface Properties

Surface topographies of the three test substrates were compared to those of a rough Ti6Al4V (rTiAlV) surface previously described by us.^15^ Disk roughness was evaluated by laser scanning microscopy (LSM710, Carl Zeiss Microscopy, LLC, Thornwood, NY). Analysis was performed over a 600 *μ*m^2^ area using a 20× objective and a cutoff wavelength of 100 *μ*m. Roughness parameters (total roughness [Rt], arithmetic average [Ra], profile roughness [Rc], maximum peak height [Rp], maximum valley depth [Rv], root-mean-squared roughness [Rq], kurtosis [Rku], skewness [Rsk]) were measured in a minimum of 20 locations on *n* = 3 disks per group. Skewness is a measurement of the lack of symmetry of a distribution probability. Positive skewness represents elevations form a relatively flat surface, whereas negative skewness represents wide plateaus eroded by deep valleys. Kurtosis is a parameter that describes the peakedness of a surface: kurtosis above 3 indicates sharp peaks, whereas kurtosis below 3 indicates rounded peaks.

### Cell Culture

Human mesenchymal stem cells (HMSCs) and normal human osteoblasts (NHOst) were obtained from Lonza Biosciences (Walkersville, MD). Cells were cultured in Mesenchymal Stem Cell Growth Media (Lonza) or Osteoblast Growth Media (Lonza), respectively, at 37 °C and 100% humidity. Cells between passage 1 and 3 were cultured on TCPS and rTiAlV as control surfaces and on each of the three test topographies at an initial cell density of 10,000 cells cm^−2^ (*n* = 6 independent cultures per substrate) and cultured in the appropriate growth medium without addition of exogenous supplements, including beta-glycerolphosphate or dexamethasone.

### Cell Morphology

Interaction of MSCs with 15 mm TiAlV disks was qualitatively examined using a cold field-emission scanning electron microscope (SEM, Hitachi Ltd., Tokyo, Japan). Secondary electron images were recorded using a 5 kV accelerating voltage and a 10 *µ*A beam current.

### Determination of Cell Response

Cultures were incubated with fresh media when cells on TCPS reached confluence, typically after 7 days. After 24 h incubation in fresh media, cultures were harvested. NHOst cells were removed from culture substrates with two sequential 10 min incubations in 0.25% trypsin, pelleted, and counted (Z2 Particle Counter, Beckman Coulter, Fullerton, CA). Cells were then lysed in 0.05% Triton X-100 and homogenized by sonication. MSC cultures were washed with PBS, lysed in 0.05% Triton X-100, and homogenized by sonication. Alkaline phosphatase specific activity [orthophosphoric monoester 3 phosphohydrolase, alkaline; E.C. 3.1.3.1] was assayed in cell lysates by measuring the conversion of *p*-nitrophenylphosphate to *p*-nitrophenol at pH 10.25, and normalized to total protein content in cell lysates. Total DNA content in MSC cultures was determined using PicoGreen (Life Technologies, Carlsbad, CA).

Conditioned media were collected and levels of secreted osteocalcin (Human Osteocalcin RIA Kit, Biomedical Technologies, Stoughton, MA), osteoprotegerin (DuoSet, R&D Systems, Minneapolis, MN), bone morphogenetic protein-2 (BMP2) (PeproTech, Rocky Mount, NJ), BMP4 (R&D Systems), vascular endothelial growth factor A (VEGFA) (R&D Systems), and fibroblast growth factor-2 (FGF2) (R&D Systems) were measured by immunoassay following manufacturer’s instructions.

Cells were harvested to measure mRNA levels after 12 h incubation in fresh media. RNA was isolated with TRIzol^®^ (Life Technologies) following manufacturer’s instructions. Equal amounts (250 ng) of RNA were reverse transcribed into cDNA for each sample (High Capacity cDNA Kit, Life Technologies). The resulting cDNA was used in real-time quantitative polymerase chain reaction (qPCR) reactions using Power SYBR^®^ Green (Life Technologies) in StepOnePlus systems (Life Technologies). mRNA quantities of integrin subunits α1, α2, α5, αv, β1, and β3 (Table 1) were calculated using standard curves generated from known dilutions of cDNA and normalized to expression of glyceraldehyde 3-phosphate dehydrogenase (GAPDH).

**Table 1:** Primer sequences used for real-time qPCR

Gene	Accession no.	Forward primer	Reverse primer
ITGA1	NM_181501.1	CACTCGTAAATGCCAAGAAAAG	TGCTTCACCACCTTCTTG
ITGA2	NM_002203	ACTGTTCAAGGAGGAGAC	TAGAACCCAACACAAAGATGC
ITGA5	NM_002205	ATCTGTGTGCCTGACCTG	GGTCAAAGGCTTGTTTAGG
ITGAV	NM_002210.2	GTTGCTACTGGCTGTTTTGG	AAG TTC CCT GGG TGT CTG
ITGB1	NM_002211	ATTACTCAGATCCAACCAC	CTGCTCCCTTTCTTGTTCTTC
ITGB3	NM_000212	AATGCCACCTGCCTCAAC	TCCTCCTCATTTCATTCATC
GAPDH	NM_002046.3	GCTCTCCAGAACATCATCC	GCGAGCACAGGAAGAAGC

### Statistical Analysis

Surface characterization was performed on *n* = 3 disks with 20 measurements taken per disk. Data from cell experiments examined six individual cultures per variable (*n* = 6). All data are presented as mean ± standard error (SE). Data were evaluated by analysis of variance, and, where differences existed, examined differences between groups using Student’s *t* test with Bonferroni’s modification for multiple comparisons. *p* < 0.05 was considered to indicate a statistically significant difference.

## Results

### Surface Analysis

Substrates used in this study contained features at the macro- (300×), micro- (10k×), submicro- (50k×), and nano-scale (100k×) (Fig. 1). The surfaces differed in their surface characteristics. At high magnification, differences in morphology at the nanoscale are evident, both with respect to the number of features and distribution but also with respect to their shape. Due to the complexity of the surface at the macro-microscale and the low profile of the nanofeatures, it was not possible to accurately quantify their dimensions.

**Figure 2:**
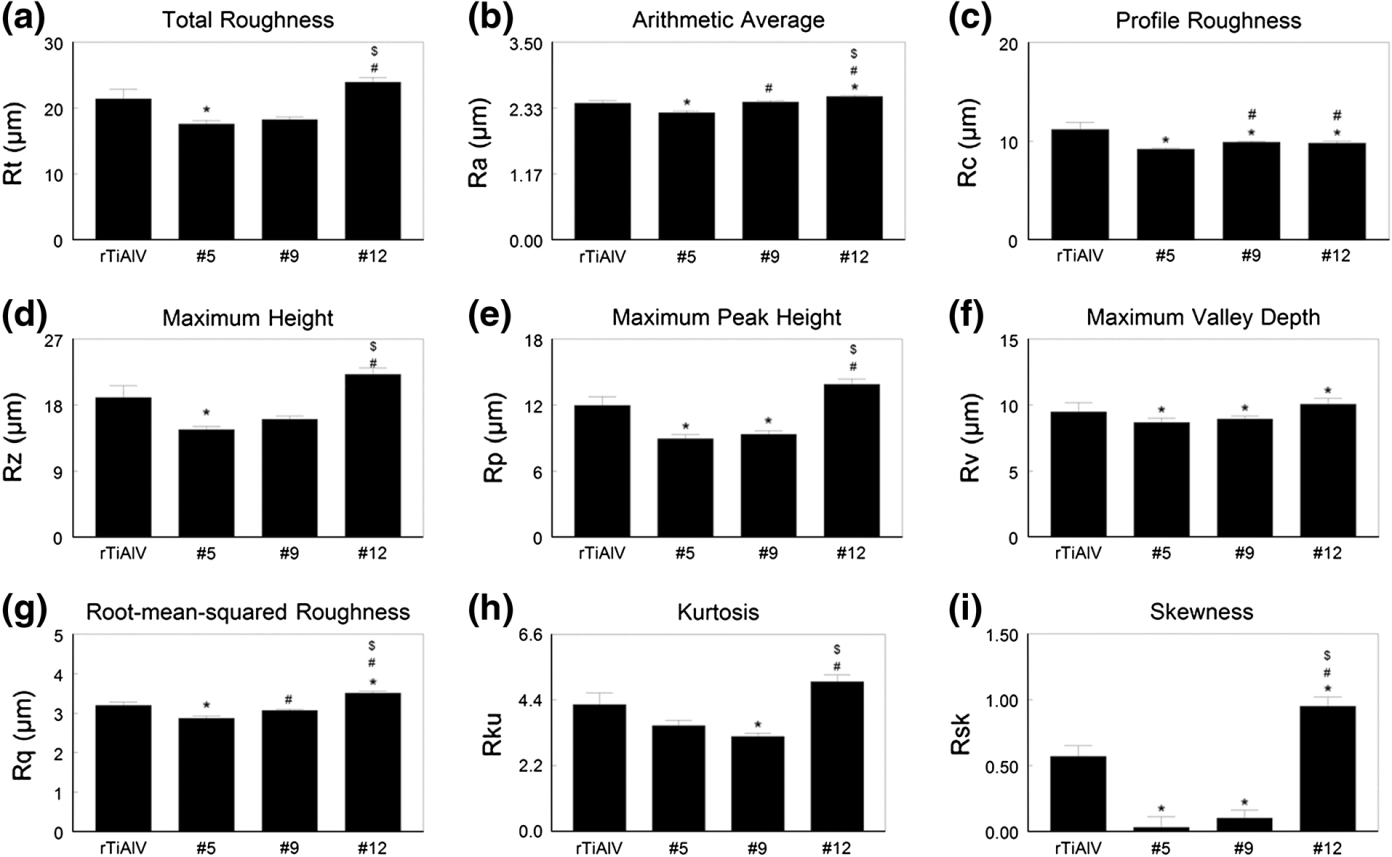
Measurement of roughness parameters. Disk roughness was characterized by laser confocal microscopy and total roughness (a), mean arithmetic roughness (b), profile roughness (c), maximum height (d), maximum peak height (e), maximum valley depth (f), root-mean-squared roughness (g), kurtosis (h), and skewness (i) measured. * *p* < 0.05 vs. rTiAlV; ^#^ *p* < 0.05 vs. #5; ^$^ *p* < 0.05 vs. #9

Total roughness (Rt) was lowest on the #5 surface and greatest on the #12 surface (Fig. 2a). Although average Ra values for #5 were lower than for the other surfaces and Ra values for #12 were highest (Fig. 2b), the actual differences in Ra were small. Profile roughness (Rc) was lower on the three test surfaces compared to the rTiAlV control (Fig. 2c). Maximum height (Rz) was lowest on #5 and highest on #12. Similarly, maximum peak height (Rp) was lowest on the #5 and #9 surfaces and highest on #12 (Fig. 2e), whereas maximum valley depth (Rv) was comparable on all surfaces (Fig. 2f). Root-mean-squared roughness (Rq) was lower on #5 and higher on #12 (Fig. 2g), kurtosis (Rku) was lowest on #9 and highest on #12 (Fig. 2h), and skewness (Rsk) was low on #5 and #9 but high on #12 (Fig. 2i).

### Cell Response

#### Osteoblasts

**Figure 3:**
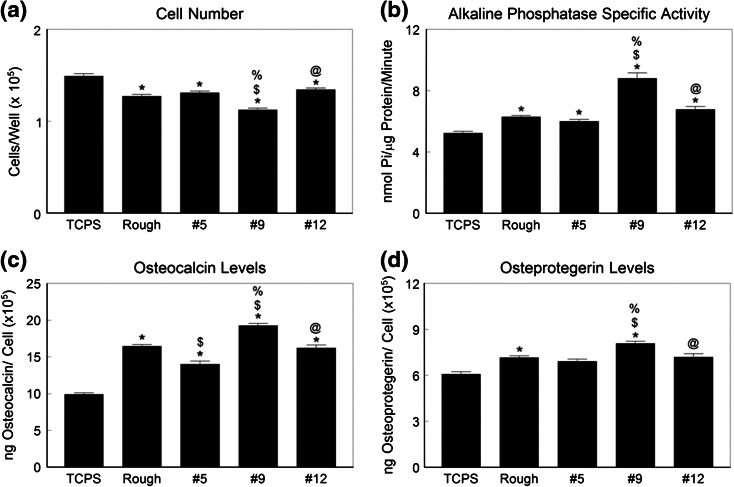
Osteoblastic maturation of normal human osteoblasts on microstructured Ti6Al4V. Osteoblasts were cultured on TCPS, rTiAlV, #5, #9, or #12 surfaces and osteoblast response measured by cell number (a), alkaline phosphatase specific activity (b), osteocalcin secretion (c), and osteoprotegerin production (d) measured. * *p* < 0.05 vs. TCPS; ^$^ *p* < 0.05 vs. rTiAlV; ^%^ *p* < 0.05 vs. #5; ^@^ *p* < 0.05 vs. #9

**Figure 4:**
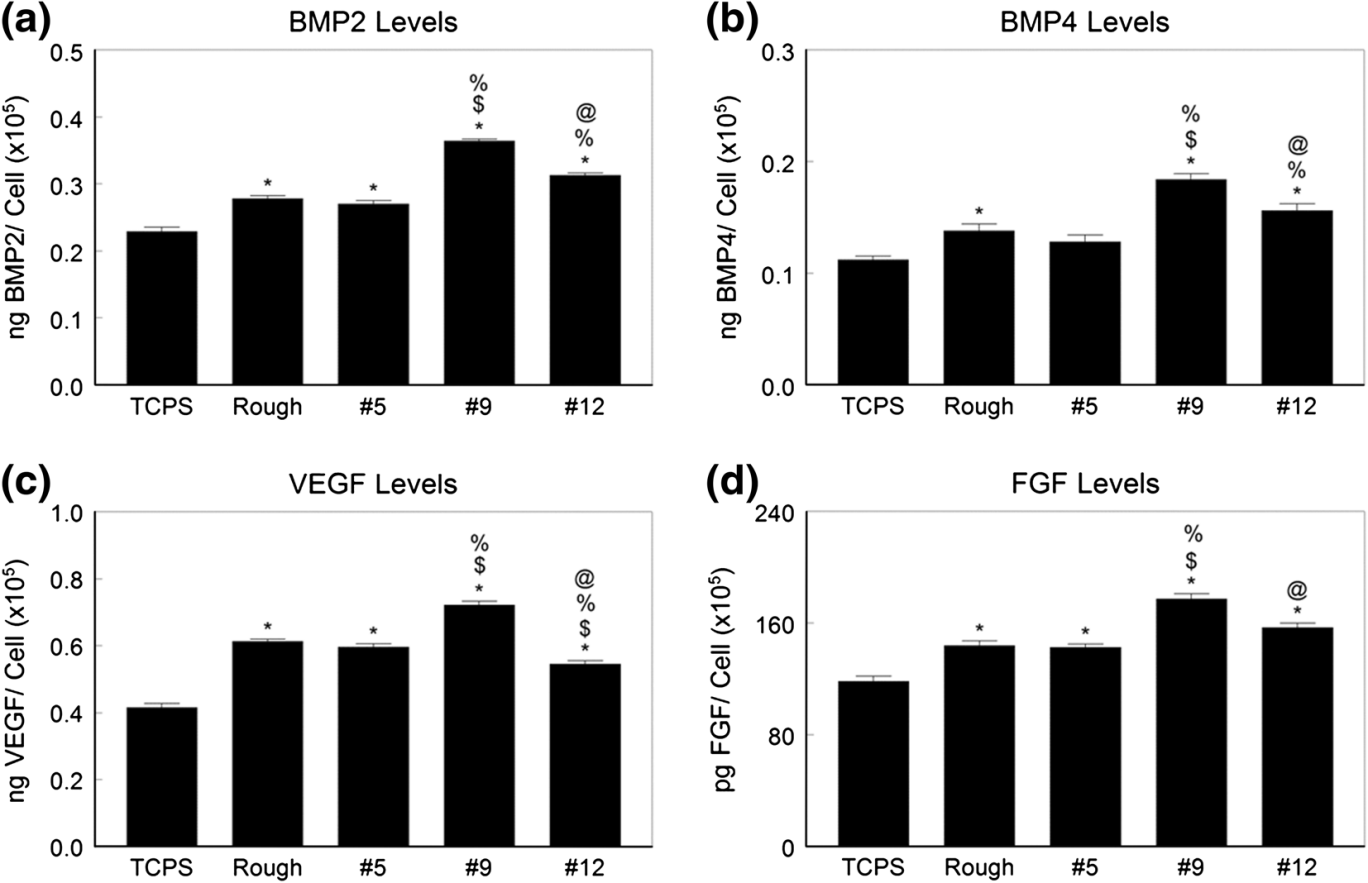
Local factor production by normal human osteoblasts on microstructured Ti6Al4V. Osteoblasts were cultured on TCPS, rTiAlV, #5, #9, or #12 surfaces and secretion of BMP2 (a), BMP4 (b), VEGFA (c), and FGF (d) measured in the conditioned media. * *p* < 0.05 vs. TCPS; ^$^ *p* < 0.05 vs. rTiAlV; ^%^ *p* < 0.05 vs. #5; ^@^ *p* < 0.05 vs. #9

Cell number was reduced on all of the Ti6Al4V substrates compared to TCPS (Fig. 3a). Surface #9 supported the lowest number of cells; all other Ti6Al4V surfaces had comparable numbers of cells. Alkaline phosphatase activity was greater on all Ti6Al4V surfaces than on TCPS and the highest enzyme activity was seen in cells grown on the #9 surface (Fig. 3b). Osteocalcin production was also sensitive to the surface topography (Fig. 3c). All test surfaces supported greater osteocalcin production than TCPS and the highest levels were seen in cultures grown on the #9 surface. Production of osteoprotegerin varied in a comparable manner (Fig. 3d). Growth factor production was sensitive to surface topography as well. BMP2 (Fig. 4a) and BMP4 (Fig. 4b) were highest in conditioned media from cultures grown on #9, as were VEGFA (Fig. 4c) and FGF2 (Fig. 4d).

**Figure 5:**
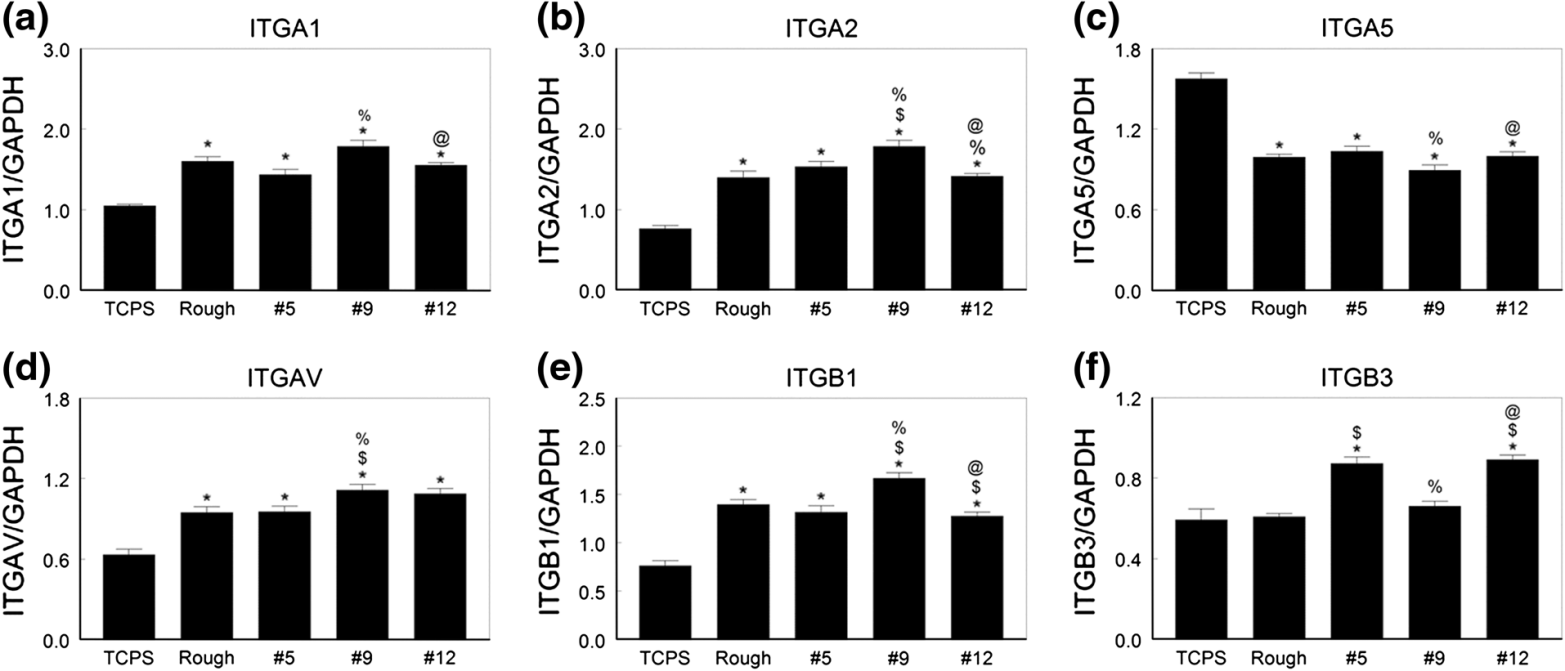
Integrin mRNA levels in normal human osteoblasts on microstructured Ti6Al4V. Osteoblasts were cultured on TCPS, rTiAlV, #5, #9, or #12 surfaces and mRNA levels of ITGA1 (a), ITGA2 (b), ITGA5 (c), ITGAV (d), ITGB1 (e), and ITGB3 (f) measured. * *p* < 0.05 vs. TCPS; ^$^ *p* < 0.05 vs. rTiAlV; ^%^ *p* < 0.05 vs. #5; ^@^ *p* < 0.05 vs. #9

Integrin expression also varied with the surface (Figs. 5a–5f). mRNAs for all integrin subunits except ITGA5 were higher when cells were cultured on Ti6Al4V substrates than on TCPS. The highest levels of mRNAs for ITGA1, ITGA2, ITGAV, and ITGB1 were in cells cultured on the #9 surface. In contrast, the lowest levels of mRNAs for ITGA5 were seen in these cells. Cells on the #12 surface also expressed higher levels of ITGAV and ITGB3 than NHOst cells cultured on the rTiAlV control surface or the #5 surface.

#### Mesenchymal Stem Cells

**Figure 6:**
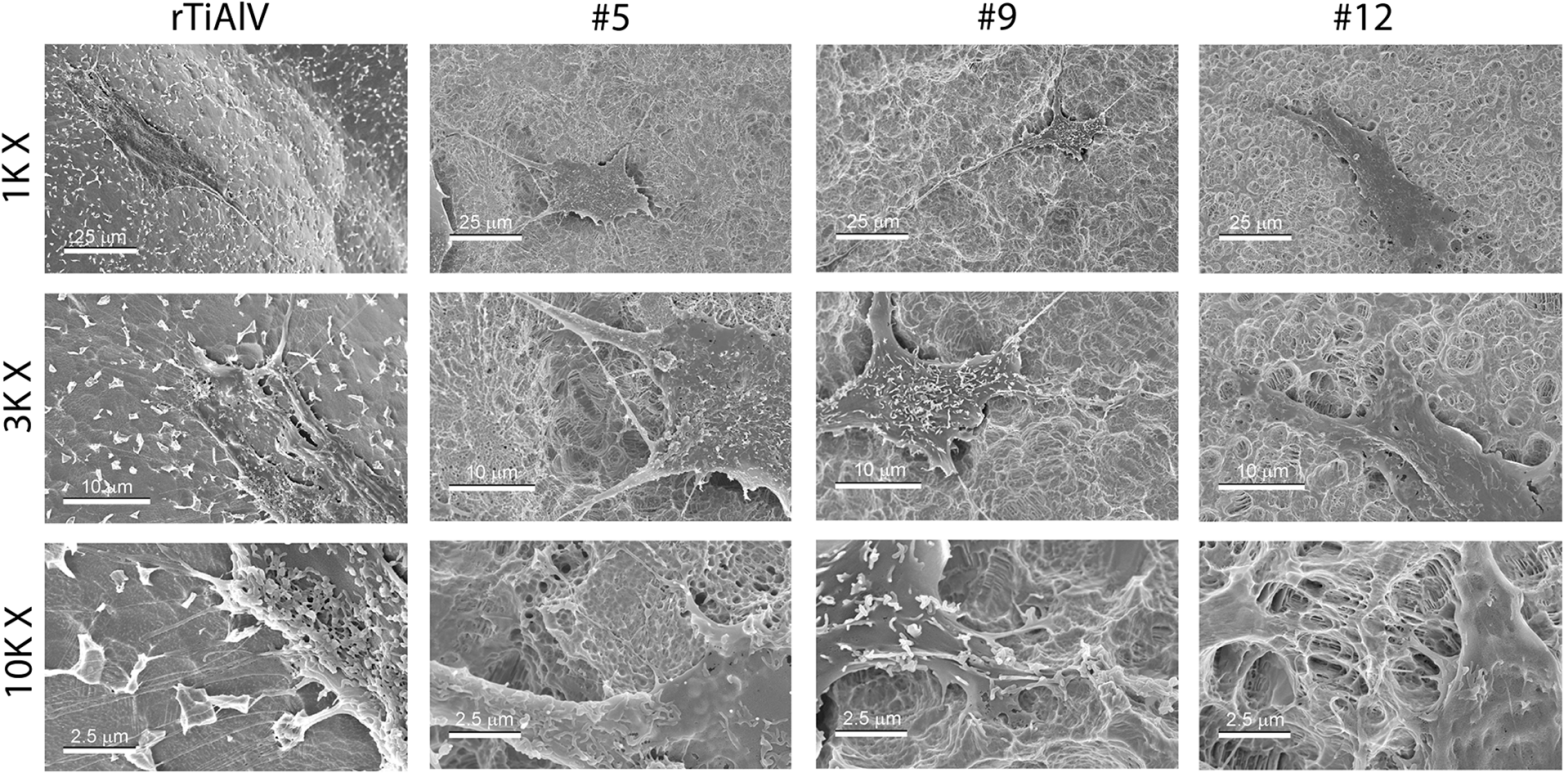
Cell morphology on microstructured Ti6Al4V. Human mesenchymal stem cells were plated on #5, #9, or #12 surfaces. Morphology of the cells was examined at low, medium, and high magnification

**Figure 7:**
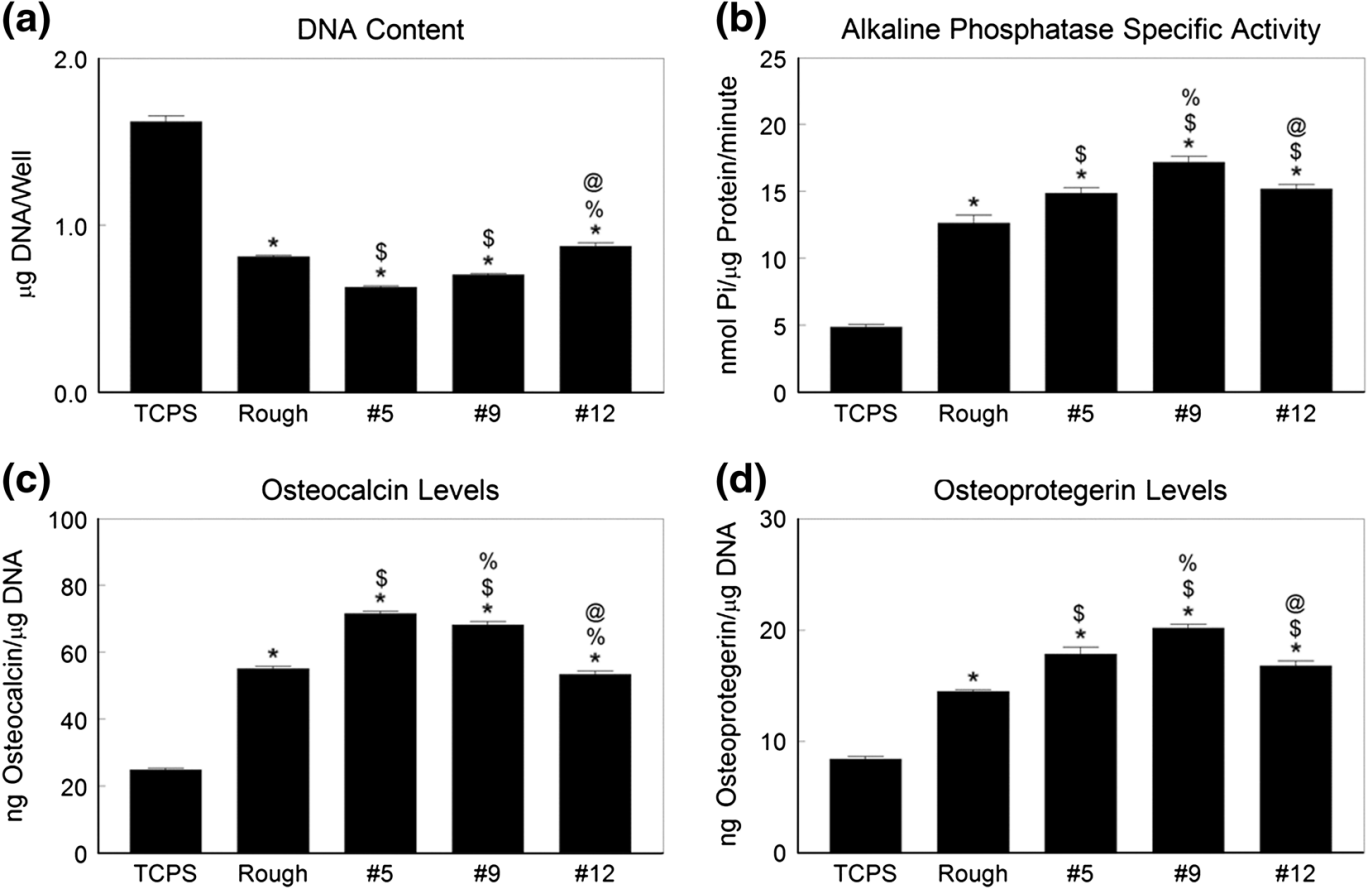
Osteoblastic differentiation of human MSCs on microstructured Ti6Al4V. HMSCs were cultured on TCPS, rTiAlV, #5, #9, or #12 surfaces and osteoblast response measured by cell number (a), alkaline phosphatase specific activity (b), osteocalcin secretion (c), and osteoprotegerin production (d) measured. * *p* < 0.05 vs. TCPS; ^$^ *p* < 0.05 vs. rTiAlV; ^%^ *p* < 0.05 vs. #5; ^@^ *p* < 0.05 vs. #9

MSCs cultured on Ti6Al4V spread well on the disk surface (Fig. 6). Cells on rTiAlV and #12 had larger bodies and smaller projections than cells on #5 and #9, which had small bodies and long projections. The number of HMSCs on the Ti6Al4V surfaces was also reduced compared to TCPS whereas markers of osteoblastic differentiation were greater on the alloy substrates. The #5 surface had the fewest HMSCs (Fig. 7a). In contrast, alkaline phosphatase specific activity was greatest in cells cultured on the #9 surface (Fig. 7b). Cells on the #5 and #9 surface produced the highest levels of osteocalcin (Fig. 7c) and HMSCs on the #9 surface produced the highest levels of osteoprotegerin (Fig. 7d).

**Fig. 8:**
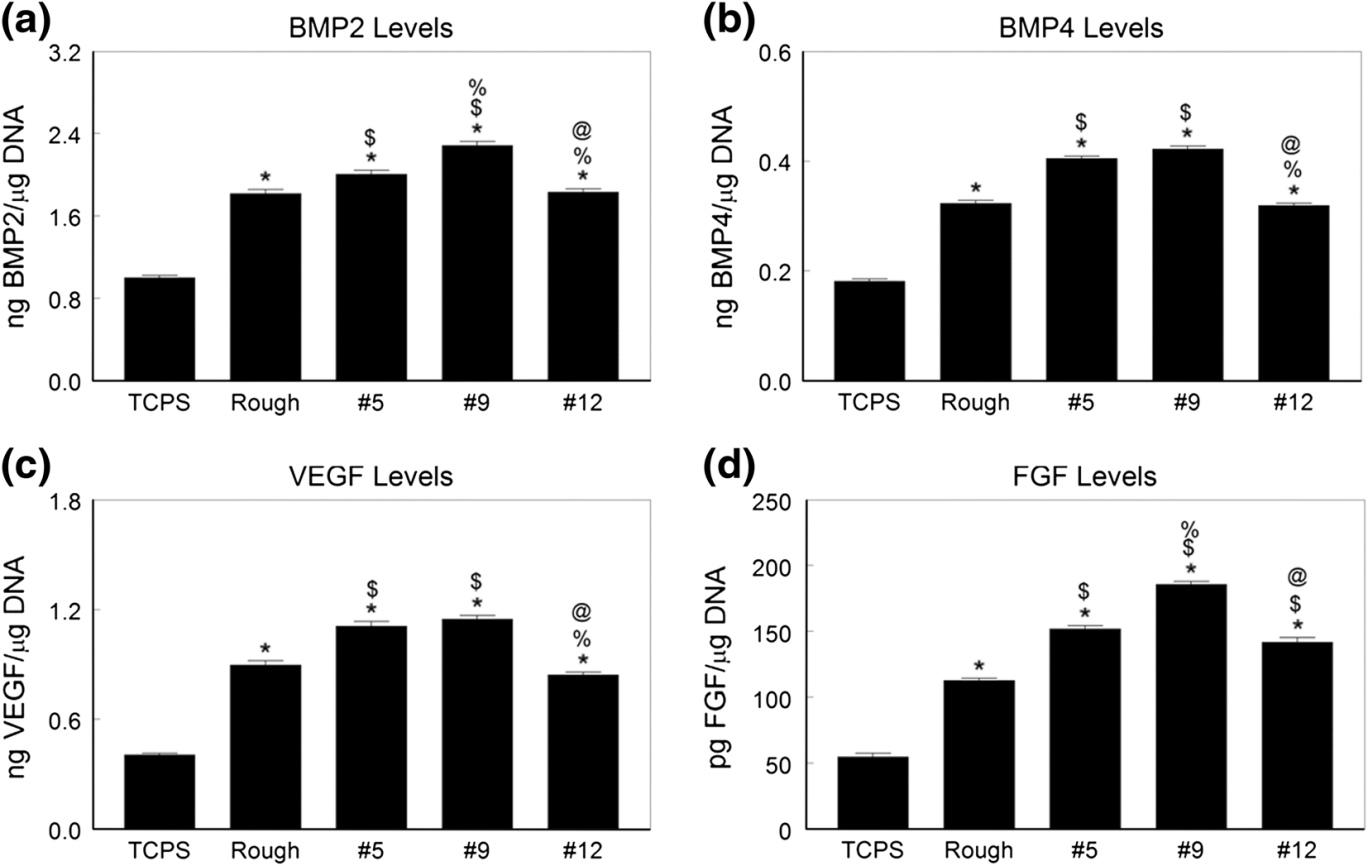
Local factor production by human MSCs on microstructured Ti6Al4V. HMSCs were cultured on TCPS, rTiAlV, #5, #9, or #12 surfaces and secretion of BMP2 (a), BMP4 (b), VEGFA (c), and FGF (d) measured in the conditioned media. * *p* < 0.05 vs. TCPS; ^$^ *p* < 0.05 vs. rTiAlV; ^%^ *p* < 0.05 vs. #5; ^@^ *p* < 0.05 vs. #9

Growth factor levels in the conditioned media were higher when MSCs were cultured on the Ti6Al4V substrates compared to TCPS. HMSCs cultured on the #5 and #9 surfaces produced more BMP2 (Fig. 8a), BMP4 (Fig. 8b) and VEGFA (Fig. 8c), than cells on the other surfaces. The highest levels of FGF2 were in conditioned media from MSCs grown on #9 (Fig. 8d).

**Figure 9:**
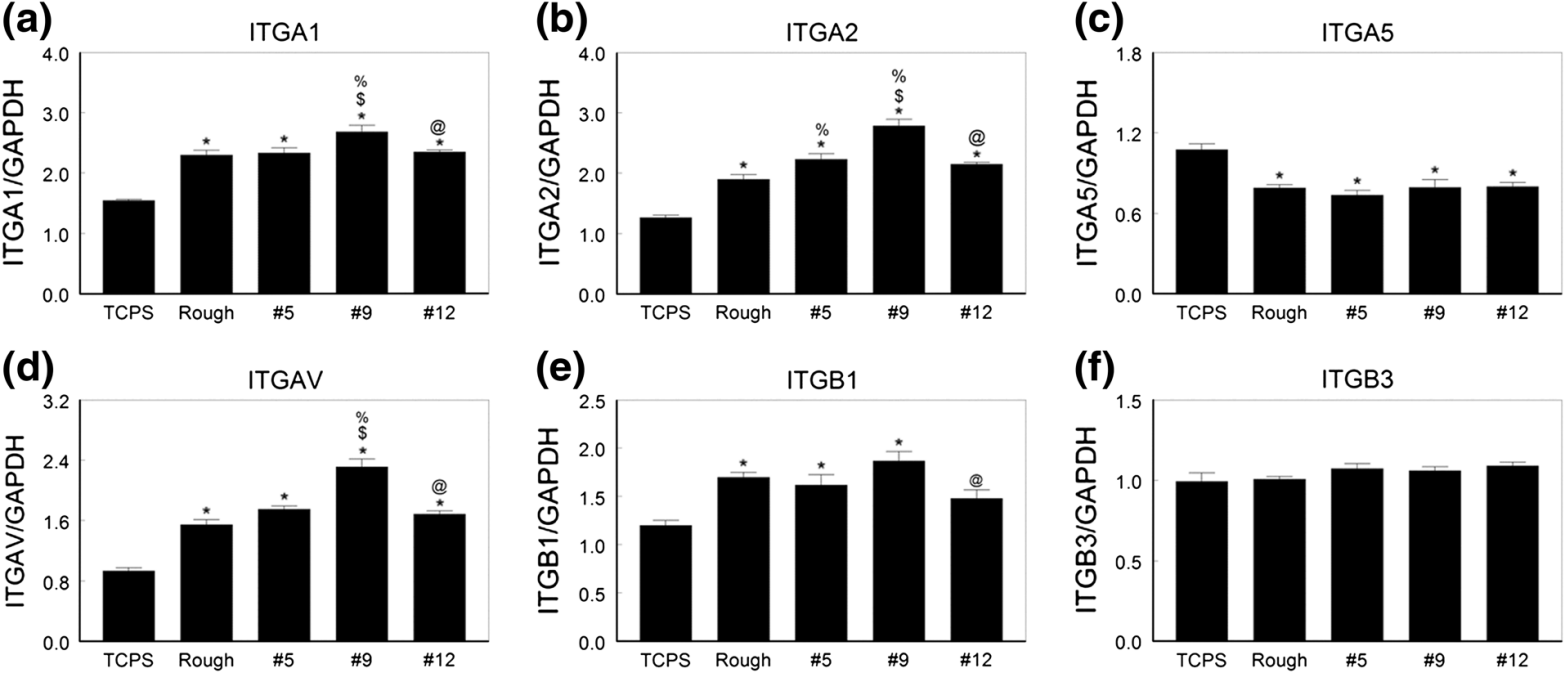
Integrin mRNA levels in human MSCs on microstructured Ti6Al4V. HMSCs were cultured on TCPS, rTiAlV, #5, #9, or #12 surfaces and mRNA levels of ITGA1 (a), ITGA2 (b), ITGA5 (c), ITGAV (d), ITGB1 (e), and ITGB3 (f) measured. * *p* < 0.05 vs. TCPS; ^$^ *p* < 0.05 vs. rTiAlV; ^%^ *p* < 0.05 vs. #5; ^@^ *p* < 0.05 vs. #9

Integrin expression was specific to the surface (Fig. 9). ITGA1 (Fig. 9a), ITGA2 (Fig. 9b), and ITGAV (Fig. 9d) were expressed at higher levels on all Ti6Al4V surfaces than on TCPS, and expression was greatest on the #9 surface. ITGA5 (Fig. 9c) was lower on all Ti6Al4V surfaces compared to TCPS and there were no differences due to surface topography. ITGB1 expression (Fig. 9e) was higher on the Ti6Al4V substrates but no surface specific differences were noted. ITGB3 (Fig. 9f) was expressed at comparable levels on all surfaces, including TCPS.

## Discussion

This study confirms our previous observations that osteoblasts exhibit a more differentiated phenotype when cultured on Ti6Al4V surfaces with a microtopography that includes submicron and nanoscale features,^15^,^18^ and expands upon our previous findings showing that nanoscale topography imposed on surfaces with rough micro- and submicro-scale topographies can enhance osteoblastic differentiation.^7^ In addition, our results confirm that HMSCs will exhibit markers consistent with osteoblast differentiation on these surfaces even in the absence of media additives commonly used to induce osteoblast differentiation on TCPS, such as beta-glycerophosphate and dexamethasone.^9^,^16^

Importantly, this study demonstrates that NHOst cells and MSCs are sensitive to specific topographical features of a microstructured surface. While the surfaces used for this study were similar in average roughness at the microscale, they differed in other topographical parameters, most notably peak height, kurtosis, and skewness. While we did not specifically quantify dimensions of the submicron and nanoscale features of the surfaces, SEMs clearly show these structural elements to be present and that differences resulted from the surface treatment. Taken together with the topographical assessments presented here, the results support the concept that the overall response of osteoblast lineage cells to an implant is modulated by its macro, micro, and nano (MMN) surface structure.

Both the #5 and #9 surfaces had lower maximum peak heights, lower kurtosis, and lower skewness than the rTiAlV or #12 surfaces. Kurtosis was most reduced on the #9 surface and skewness was most reduced on the #5 surface. These results correlated with greater osteoblastic differentiation of both cell types on the #9 surface and with greater production of growth factors associated with osteogenesis *in vivo*. Taken together the results indicate that the optimal microtextured Ti alloy surface may have an average Ra between 2 and 3 *µ*m, but with moderate peak height, low kurtosis, and slight skewness. Pull out strength of commercially pure Ti implants also has been shown to correlate with skewness and kurtosis.^14^ In contrast to our observations, these investigators did not observe a correlation with average roughness. Whether this reflects differences in other surface properties, including chemistry and macro, micro, and nanoscale features, is not known.

Our results also show that integrin expression in committed osteoblasts and MSCs differs in a surface dependent manner. We previously reported that human osteoblast-like MG63 cells express higher levels of ITGA2 and ITGB1 on rTiAlV than on TCPS.^18^ Moreover, differentiation of osteoblasts requires signaling by α2β1.^19^ mRNAs for ITGA2 and ITGB1 were highest on the #9 surface, suggesting that the greater osteoblastic differentiation noted on this surface resulted from signaling *via* this integrin. Levels of ITGA1 were also elevated on #9. Like α2β1, the α1β1 integrin binds collagen,^6^ indicating the importance of the interaction with type I collagen in determining cell fate.

Although expression of ITGAV was elevated in NHOst cells on the #9 surface, mRNAs for its partner ITGB3 were not increased compared to TCPS. Moreover, mRNAs for ITGA5 were reduced compared to TCPS and to the other Ti6Al4V surfaces. This suggests that the role played by these integrins in osteoblastic differentiation on microstructured Ti6Al4V surfaces is either very small or had already occurred prior to the time of measurement. ITGB3 mRNA levels were markedly increased on #5 and #12, but the meaning of this is not clear at this time. The αvβ3 integrin pair binds vitronectin,^3^ which may be an important extracellular protein for cells on these surfaces.

Osteoblastic differentiation of HMSCs was also increased on the Ti6Al4V substrates in a surface dependent manner. Differentiation on the three test surfaces was either comparable to the rTiAlV control surface or greater than seen on rTiAlV. As noted for NHOst cells, HMSCs exhibited high alkaline phosphatase and osteocalcin on the #9 surface, but cells on the #5 surface also exhibited increased osteoblastic differentiation compared to #12, supporting the hypothesis that kurtosis and skewness are important variables. This was the case for growth factor production as well. The observation that HMSCs produced the highest levels of osteoprotegerin and FGF2 when cultured on #9 suggests that this surface has potential to generate greater peri-implant osteogenesis, including greater bone formation vs. resorption and greater angiogenesis. Whether this is the case requires *in vivo* assessment of peri-implant bone formation.

As noted for NHOst cells, expression of ITGA1 and ITGA2 mRNAs was greatest on the #9 surface, supporting a role for collagen in determining cell fate. ITGA5 expression was reduced in all cultures grown on Ti6Al4V substrates, confirming the reduced role for α5β1 in osteoblastic differentiation compared to TCPS.^12^ Unlike NHOst cells, however, HMSCs produced higher ITGAV mRNAs on #9 than on all other surfaces, but expression of mRNA for its partner β3 was not sensitive to the surface type.

## Conclusions

Overall, these results demonstrate clearly that surface properties in addition to chemistry and average roughness are important variables in determining how cells in the osteoblast lineage will respond to implants. While many commercial implants incorporate macroscale roughened surfaces for mechanical stability during arthrodesis, these features are too large to affect the healing response at a cellular level. By examining surfaces with similar average roughness, these results show that both committed osteoblasts and multipotent MSCs can discriminate surface features at the microscale and are particularly sensitive to kurtosis and skewness, suggesting that they are sensitive to nanoscale features as well.

While statistically significant differences were found with respect to cell response, this study did not determine if they were correlated with a significant difference in clinical outcome. We have previously shown that osteoblasts can detect differences in submicron scale topographic features on Ti substrates.^27^ Moreover, topographical differences can result in differences in bone-to-implant contact and pullout strength *in vivo*.^22^ There is a growing body of literature indicating that cells can detect nanoscale differences when grown on TCPS surfaces.^4^,^25^ Given that TCPS is not a clinically relevant material the observations presented here are of particular importance in defining what surface properties are most valuable in the design of implants used in musculoskeletal applications.
